# Diagnostic accuracy and clinical relevance of an inflammatory biomarker panel in early sepsis in adult critical care patients

**DOI:** 10.1186/cc14135

**Published:** 2015-03-16

**Authors:** P Bauer, R Kashyap, S League, J Park, D Block, N Baumann, A Algeciras-Schimnich, S Jenkins, C Smith, O Gajic, R Abraham

**Affiliations:** 1 Mayo Clinic, Rochester, MN, USA

## Introduction

Low awareness, late recognition and delayed treatment of sepsis are still common. CD64 is a marker of the innate immune response upregulated in sepsis. The primary goal of this prospective, double-blind study was to compare the diagnostic accuracy of neutrophil CD64 and other cellular markers, along with C-reactive protein (CRP) and procalcitonin (PCT) levels, in early sepsis.

## Methods

Adult ICU patients, between 2012 and 2014 were eligible. The eight-color flow cytometric biomarker panel included CD64, CD163, HLA DR, CD15 and others. Diagnostic test results were compared with infection as the reference standard and sepsis as the target condition, using receiver operating characteristic curve analyses. Multivariable logistic regression was used to assess the relationship of sets of markers with the probability of sepsis, adjusting for other patient characteristics.

## Results

A total of 219 patients were enrolled, 120 with sepsis, 99 served as controls. APACHE IV (median 70 vs. 57), SOFA (8 vs. 7), ICU (2 vs. 1) and hospital length of stay (6 vs. 4) were higher in the sepsis group. Mortality was not different. After adjustment for APACHE IV, CRP and PCT, CD64 molecules/neutrophil measure remained a significant predictor of sepsis (OR = 1.852 for 1-unit increase on the log scale, 95% CI = 1.083 to 3.168, *P *= 0.02, AUC = 0.90). See Table 1.

**Table 1:** Area under the curve (AUC) for individual biomarkers

**Measure**	***N***	**AUC**	**Cutoff for sepsis**	**Sensitivity (%)**	**Specificity (%)**
C-reactive protein (mg/l)	208	0.86	43	76.9	76.9
CD64 molecules/neutrophil	196	0.83	1,040.5	76.4	76.7
Procalcitonin (ng/ml)	216	0.82	0.74	73.1	73.2
%CD64^+ ^neutrophils	196	0.81	49.96	74.5	74.4

## Conclusion

Neutrophil CD64 expression is an accurate predictor of early sepsis.

